# Rhizosphere Microbiota and Soil Nutrients Shape Fruit Lignan Composition of *Schisandra chinensis* Across Temperate Cultivation Sites in Northeast and Northwest China

**DOI:** 10.3390/life15101555

**Published:** 2025-10-03

**Authors:** Yanli Wang, Wenpeng Lu, Jiaqi Li, Yiming Yang, Shutian Fan, Yue Wang, Hongyan Qin, Nan Shu, Baoxiang Zhang, Changyu Li, Jingmeng Zhu, Jinshuo Wang, Sisi Yang, Peilei Xu

**Affiliations:** 1 Institute of Special Animal and Plant Sciences, Chinese Academy of Agricultural Sciences, Changchun 130112, China; wangyanli@caas.cn (Y.W.); luwenpeng@caas.cn (W.L.); lijiaqi@caas.cn (J.L.); yym0312@163.com (Y.Y.); fanshutian@caas.cn (S.F.); y1989w@126.com (Y.W.); qinyan11@163.com (H.Q.); shunan_caas@163.com (N.S.); zbx0319@126.com (B.Z.); lichangyu@caas.cn (C.L.); 821012430257@caas.cn (J.Z.); 2 College of Food Science, Jilin Agricultural University, Changchun 130118, China; 3 College of Agricultural Sciences, Yanbian University, Yanji 133002, China; 18363581226@163.com; 4 Institute of Science and Technology information of Jilin Province, Changchun 130033, China

**Keywords:** correlation analysis, diversity, lignans, rhizosphere microbial, *Schisandra chinensis*

## Abstract

*Schisandra chinensis* (Turcz.) Baill. (S. *chinensis*) is a widely used medicinal plant whose therapeutic efficacy is closely linked to its lignan content. While previous studies have focused on soil fertility and cultivar variation, the interplay among soil nutrients, rhizosphere microbiota, and lignan accumulation remains poorly understood. This study investigated *S. chinensis* grown across 20 cultivation sites to elucidate the relationships among soil nutrient profiles, fruit lignan composition, and rhizosphere microbial communities. Six major lignans were quantified using HPLC, soil nutrients were analyzed via standard chemical assays, and rhizosphere bacterial communities were profiled using 16S rRNA sequencing. Multivariate analyses revealed significant variation in soil properties and lignan content across sites. Notably, available phosphorus, organic matter, and total nitrogen showed strong correlations with specific lignan compounds. From the top 50 taxa ranked by relative abundance at the genus level, 18 bacterial genera associated with lignan components were identified. Among them, *Mycobacterium*, *Arthrobacter*, *Haliangium*, *Bacillus*, *Sphingomonas*, *Rhodanobacter*, *Ellin6067*, *Bradyrhizobium*, *Pseudolabrys*, *Chujaibacter*, *Gemmatimonas*, *Bryobacter*, *MND1*, *Candidatus Sollbacter*, *Gaiella*, *Paenibacillus*, *RB41*, and *Candidatus_Udaeobacter* were significantly associated with lignan levels, suggesting potential microbial involvement in lignan biosynthesis. These findings provide insights into the ecological factors shaping the medicinal quality of *S. chinensis* and offer a foundation for targeted cultivation and breeding strategies.

## 1. Introduction

*Schisandra chinensis* (Turcz.) Bail. (*S. chinensis*) is a plant of the Schisandraceae family, is also known as mountain pepper, and is commonly called “Northern *Schisandra chinensis*”. It is mainly distributed in Northeast China, the Korean Peninsula, and the Far East of Russia [1]. It has a long history of medicinal use, which can be traced back to the “Compendium of Materia Medica” compiled by Li Shizhen in 1596. It has the effects of astringing and securing, replenishing qi and promoting the production of body fluids, tonifying the kidney and calming the mind, etc. [2]. Modern pharmacological studies have shown that *S. chinensis* is rich in various bioactive components, including lignans, phenolic acids, triterpenoids, polysaccharides, volatile oils, and vitamins [3]. Among them, lignans, as genus-specific polyphenolic compounds, are its most representative pharmacologically active components [4]. To date, 86 types of lignans have been isolated and identified from *S. chinensis*. In particular, dibenzocyclooctadiene lignans have shown significant activities in liver protection, anti-inflammatory, anti-oxidation, anti-cancer, and other aspects [5,6,7,8,9,10]. In addition, the fruits of *S. chinensis* are also rich in functional components such as organic acids, anthocyanins, and vitamins C and E, making them widely used in the fields of traditional Chinese medicine, health products, and cosmetics [11,12]. With the expansion of the application fields of *S. chinensis*, the industry of *S. chinensis* Chinese medicinal materials has shown a strong development momentum, and its planting scale has been expanding year by year. However, there is a significant difference in the quality of *S. chinensis* [13]. Studies have reported that compared with wild *S. chinensis* resources, the active ingredients of field-grown *S. chinensis* are lower, while the active ingredients of understory-grown *S. chinensis* are close to those of the wild type. Therefore, it is imperative to study the cultivation techniques of *S. chinensis* and improve its medicinal quality [14,15]. Therefore, it is imperative to study the cultivation techniques of *S*. *chinensis* and improve its medicinal quality. Up to now, a large number of research reports have still focused on the study of the pharmacological activities of *S*. *chinensis* [16,17,18,19,20,21,22,23]. Research on its cultivation techniques remains relatively superficial, and there is still a lack of systematic exploration on the relationships between the soil environment, rhizosphere microbial community, and the active components of *S*. *chinensis* [13]. Existing studies have shown that plant rhizosphere microorganisms play an important role in regulating the synthesis of secondary metabolites, and soil physical and chemical properties may also affect the accumulation of medicinal components [24,25].

Given the observed variation in fruit lignan content across cultivation regions, we hypothesize that soil nutrient profiles play a critical role in regulating lignan accumulation in *Schisandra chinensis* and that site-specific differences in rhizosphere microbial communities are closely linked to lignan biosynthesis. To test these hypotheses, we conducted a comparative study using wild germplasm resources from Zhashui (Shaanxi) and Jingyu (Jilin), analyzing soil physicochemical properties, rhizosphere microbial composition, and fruit lignan profiles. Although previous studies have suggested that soil conditions and microbial communities can influence the production of secondary metabolites in medicinal plants, the integrated mechanism connecting soil–microbe–metabolite interactions in *S. chinensis* remains largely unexplored. This study aims to elucidate how environmental factors shape the medicinal quality of *S. chinensis*, thereby providing a scientific foundation for breeding high-quality varieties, developing ecological cultivation strategies, and promoting the sustainable development of the *S. chinensis* industry.

## 2. Materials and Methods

### 2.1. Collection of Plant and Soil Samples

The experimental materials in this study consisted of rhizosphere soil and mature fruit samples from 20 individual *S. chinensis* plants. Samples Sc1–Sc14 were collected from naturally grown plants in Jingyu County, Jilin Province, while Sc15–Sc20 were obtained from cultivated plants in Zhashui County, Shaanxi Province. For each plant, three subsamples were collected as replicates. Detailed sampling locations and geographic coordinates are provided in Table 1. For each site, healthy and vigorously growing *S. chinensis* plants, free from visible signs of disease or insect infestation, were selected for sampling.

During the harvest season, mature fruits of *S. chinensis* were collected, thoroughly rinsed with deionized water, and dried in a forced-air drying oven (DHG-9013A, Shanghai Yiheng Scientific Instruments Co., Ltd., Shanghai, China). The dried fruits were then ground into powder using a laboratory grinder (TQ-500Y, Yongkang Tianqi Shengshi Industry and Trade Co., Ltd., Jinhua, China). To obtain rhizosphere soil samples, surface weeds surrounding the selected plants were carefully removed, and the root systems were gently excavated. Loose soil was shaken off, and the soil tightly adhering to the root surfaces was meticulously brushed off using sterile brushes. The collected rhizosphere soil was transferred into sterile 2 mL centrifuge tubes, flash-frozen in liquid nitrogen, and stored at −80 °C until further analysis.

### 2.2. Detection of Soil Physicochemical Properties

Soil pH was measured using a calibrated pH meter after mixing soil and water in a ratio of 1:2.5 [26].

Organic matter (OM) content was quantified via the potassium dichromate volumetric method with external heating [26].

Total phosphorus (TP) was determined by sulfuric acid–perchloric acid (H_2_SO_4_-HClO_4_) digestion. Specifically, 1.0000 g of air-dried soil (passed through a 0.149 mm sieve) was digested with 4 mL of concentrated H_2_SO_4_ and 1 mL of HClO_4_ on a heating plate until the sample turned grayish-white. After cooling, the digest was diluted to 100 mL with distilled water and analyzed using a continuous flow chemical analyzer (SKALAR SAN++) [27].

Available phosphorus (AP) was extracted using 0.5 M sodium bicarbonate (NaHCO_3_). A 1.25 g soil sample (2 mm sieve) was shaken with 25 mL of extractant for 30 min at room temperature. The filtrate was analyzed using the SKALAR SAN++ system [27].

Total potassium (TK) was analyzed using inductively coupled plasma atomic emission spectrometry (ICP-AES, ICPS-7500). A 0.1000 g soil sample (0.149 mm sieve) was digested with a mixture of HNO_3_, HClO_4_, and HF. After digestion and evaporation to near dryness, the residue was diluted with 10% HNO_3_ and analyzed. A blank experiment was conducted in parallel [27].

Available potassium (AK) was extracted using 1 M ammonium acetate (CH_3_COONH_4_). A 2.50 g soil sample (2 mm sieve) was shaken with 25 mL of extractant for 30 min and filtered. The filtrate was analyzed using ICP-AES (ICPS-7500) [27].

Total nitrogen (TN) was determined using the SKALAR SAN++ analyzer. A 1.0000 g soil sample (0.149 mm sieve) was digested with 1.8 g of catalyst mixture (Se:CuSO_4_:K_2_SO_4_ = 1:10:100) and 4 mL of concentrated H_2_SO_4_ until the solution turned greenish and no black particles remained. The digest was diluted to 100 mL and analyzed [26,27].

Ammonium Nitrogen (AN) was Measured using the diffusion-titration method. A 2.00 g soil sample (2 mm sieve) was placed in the outer chamber of a diffusion dish. The inner chamber contained 3 mL of 2% boric acid (H_3_BO_3_) indicator. After sealing, 10 mL of 1 mol/L NaOH was added, and the dish was incubated at 40 °C for 24 h. The boric acid solution was then titrated with 0.012 mol/L HCl until the endpoint color changed. A blank was run simultaneously for calibration [26,27].

### 2.3. Determination of Schisandra Lignan Content

Standard reference solutions were prepared by accurately weighing 1.00 mg of schisandrol A (CJ), (National Institutes for Food and Drug Control, Beijing, China, Batch No. 110857-201412), 1.43 mg of schisandrol B (CY), (Shanghai Ronghe Pharmaceutical Technology Co., Ltd., Shanghai, China, Batch Number 151024), 1.24 mg of schisantherin A (ZJ), (Shanghai Ronghe Pharmaceutical Technology Co., Ltd., Shanghai, China, Batch Number 151103), 1.25 mg of schisandrin A(JS), (National Institutes for Food and Drug Control, Beijing, China, Batch No. 110764-201513), 1.29 mg of schisandrin B(YS), (National Institutes for Food and Drug Control, Beijing, China, Batch No. 110765-201311) and 1.16 mg of schisandrin C(BS), (Shanghai Ronghe Pharmaceutical Technology Co., Ltd., Shanghai, China, Batch No. 151009). Each compound was dissolved in HPLC-grade methanol (Thermo Fisher China Co., Ltd., Beijing, China, UN1230) and diluted to a final volume of 5 mL. The solutions were vortex-mixed and subsequently filtered through a 0.45 μm organic membrane filter prior to use.

Dried *S. chinensis* fruit powder (0.10 g per sample) was accurately weighed in six replicates and transferred into 15 mL centrifuge tubes. Each sample was extracted with 10 mL of HPLC-grade methanol. The mixtures were vortexed for 20 min, incubated in a water bath at 65 °C for 20 min, and then subjected to ultrasonic extraction using a 320 W generator (Shenzhen Jiemeng Cleaning Equipment Co., Ltd., Shenzhen, China, JP-010T) at 65 °C for an additional 20 min. The resulting extracts were filtered through a 0.45 μm membrane into vials for chromatographic analysis.

Lignan separation was performed using an Agilent 880975-902 SB-C18 analytical column (4.6 × 250 mm). The mobile phase consisted of methanol (solvent D) and water (solvent C), applied under a gradient elution program as detailed in Table 2. The flow rate was maintained at 1.0 mL/min, the column temperature was set to 35 °C, and detection was carried out at 220 nm. The injection volume was 10 μL.

### 2.4. Assessment of Microbial Diversity in Rhizosphere Soil

Microbial DNA was extracted from rhizosphere soil samples using the E.Z.N.A.^®^ Soil Microbial DNA Extraction Kit (Omega Bio-Tek, Norcross, GA, USA). The V3–V4 hypervariable region of the bacterial 16S rRNA gene was amplified using primers 338F (5′-ACTCCTACGGGAGGCAGCAG-3′) and 806R (5′-GGACTACHVGGGTWTCTAAT-3′). Purified amplicons were sequenced on the Illumina MiSeq PE300 platform [28]. Raw sequencing data were deposited in the National Genomics Data Center (NGDC), (https://ngdc.cncb.ac.cn/gsub/submit/bioproject/PRJCA045516) (accessed on 1 September 2025). All sequencing work was performed by Majorbio Bio-Pharm Technology Co., Ltd. (Shanghai, China).

Sequence data were processed using a combination of internal Perl scripts, fastp (v0.19.6) [29], and FLASH (v1.2.7) [30] for quality filtering, merging, and optimization. High-quality sequences were clustered into operational taxonomic units (OTUs) at a 97% similarity threshold using UPARSE (v7.1) [31,32]. Taxonomic classification and annotation were performed using the RDP Classifier Bayesian algorithm [33]. Bioinformatics analyses were conducted on the MeijiYun cloud platform. Alpha diversity indices were calculated using Mothur (v1.30.1) [34] based on OTU data. Differences in microbial community composition among samples were assessed using R (v3.3.1) and the pheatmap package (v1.0.8). Community similarity was evaluated using the Vegan package (v2.5.3), and correlation analyses were also performed using pheatmap.

### 2.5. Data Analysis

Statistical analyses of the mean values, standard deviations, and multiple comparisons (Tukey’s HSD test) for soil physicochemical properties and schisandrin content in *S. chinensis* were performed using SPSS 23.0 software. Spearman’s correlation analysis was conducted using GraphPad Prism 8.

## 3. Results

### 3.1. Physicochemical Characteristics of Rhizosphere Soil of S. chinensis

Eight physicochemical parameters were analyzed across twenty rhizosphere soil samples collected from *S. chinensis* cultivation sites (Table 3). The soils exhibited slightly acidic conditions, with pH values ranging from 5.27 to 6.46, which are generally conducive to plant growth [35]. Significant differences were observed in the contents of soil organic matter (OM), available phosphorus (AP), available potassium (AK), and total nitrogen (TN) among the soil samples. OM ranged from 2.84% to 24.55%, with a coefficient of variation (CV) of 59.54%. AP varied between 10.03 mg/kg and 299.16 mg/kg (CV = 93.48%), while AK ranged from 32.28 mg/kg to 244.3 mg/kg (CV = 56.35%). TN concentrations spanned from 1395.72 mg/kg to 11,371.56 mg/kg, indicating substantial variability in nitrogen availability. Further analysis revealed that samples from Zhashui exhibited TN levels between 1395.72 and 1981.05 mg/kg, whereas samples from Jingyu ranged from 3466.88 to 11,371.56 mg/kg—representing a 1.8- to 8.1-fold increase compared to Zhashui. Similarly, available nitrogen (AN) in Zhashui samples ranged from 114.24 to 146.72 mg/kg, while Jingyu samples showed markedly higher values between 266.56 and 688.24 mg/kg, which were 1.8 to 6.0 times greater than those in Zhashui.

These findings highlight substantial spatial heterogeneity in soil nutrient distribution across sampling sites. Such variability may significantly influence the growth performance of *Schisandra chinensis* and the biosynthesis of its pharmacologically active compounds.

### 3.2. Variation in Lignan Content Among Schisandra chinensis Fruit Samples

The lignan content in *S*. *chinensis* fruit samples is summarized in Table 4, with each value representing the mean ± standard deviation from three replicate measurements. Among the 20 samples, schisandrol A (CJ) ranged from 4.34 to 9.11 mg/g, with a coefficient of variation (CV) of 22.11%, indicating relatively low variability and no significant differences among samples. In contrast, schisantherin A (ZJ) showed a wider range of 0.40 to 2.24 mg/g and a CV of 57.84%; schisandrin B (CY) ranged from 0.67 to 6.96 mg/g (CV = 56.01%); and schisandrin C (BS) varied from 0.13 to 1.37 mg/g (CV = 68.32%). These components exhibited substantial variation across samples, suggesting significant differences in their accumulation patterns.

### 3.3. Correlation Analysis Among Soil Physicochemical Properties and Lignan Components

**Figure 1:**
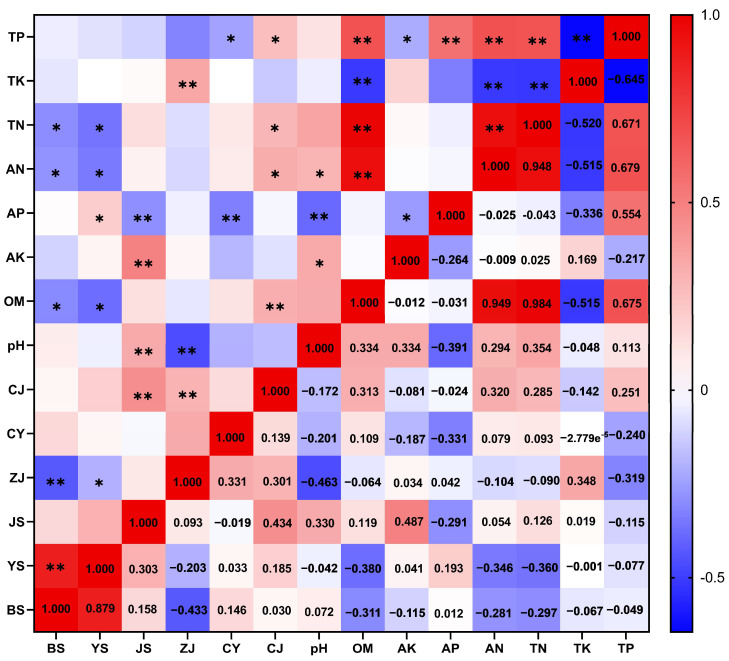
Spearman correlation analysis of soil physical and chemical indicators and lignans (Spearman’s ρ). TP: total phosphorus; TK: total potassium; TN: total nitrogen; AN: available nitrogen; AP: available phosphorus; AK: available potassium; OM: soil organic matter; pH: soil pH; CJ: Schisandrol A; CY: Schisandrol B; ZJ: Schisantherin A; JS: Schisandrin A; YS: Schisandrin B; BS: Schisandrin C. TL: total lignans. The lower right triangle displays Spearman’s correlation coefficients (ρ), while the upper left triangle indicates statistical significance: * denotes *p* < 0.05, ** denotes *p* < 0.01.

Spearman’s correlation analysis (ρ) between soil physicochemical indicators and lignan content was conducted using GraphPad Prism 8 software (Figure 1). The results revealed that total potassium (TK) exhibited extremely significant negative correlations with organic matter (OM), available phosphorus (AP), alkali-hydrolyzable nitrogen (AN), and total nitrogen (TN). Available potassium (AK) was significantly positively correlated with soil pH. Available phosphorus (AP) showed significant negative correlations with both pH and AK. Total phosphorus (TP) was significantly positively correlated with OM, AP, TN, and AN but significantly negatively correlated with TK (Figure 1).

Correlation analysis revealed distinct relationships between lignan content and soil physicochemical parameters. Samples with higher levels of schisandrol A (CJ) tended to exhibit elevated concentrations of organic matter (OM), total phosphorus (TP), and total nitrogen (TN). In contrast, higher schisandrol B (CY) content was associated with lower levels of available phosphorus (AP). Schisantherin A (ZJ) showed a positive correlation with total potassium (TK), but a negative correlation with both pH and TP. Schisandrin A (JS) content increased with higher levels of available potassium (AK) and pH, while it decreased with increasing AP. Notably, schisandrin B (YS) and schisandrin C (BS) contents were negatively correlated with TN, alkali-hydrolyzable nitrogen (AN), and OM (Figure 1).

### 3.4. Analysis of Microbial Diversity in the Rhizosphere of S. chinensis

**Figure 2:**
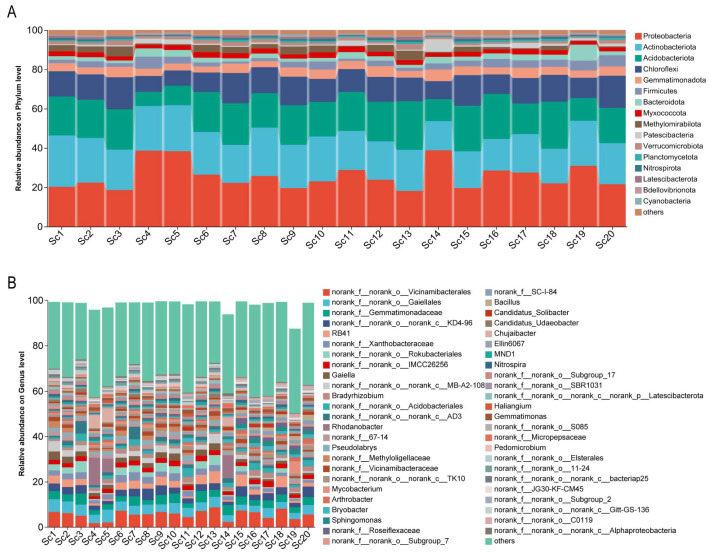
Community barplot analysis of rhizosphere bacterial composition associated with different *S. chinensis* resources. (**A**) Relative abundance of rhizosphere bacterial communities at the phylum level. (**B**) Relative abundance of rhizosphere bacterial communities at the generic level. Sc1-Sc20: *S. chinensis* at different cultivation sites.

To investigate the microbial diversity in the rhizosphere of *S*. *chinensis*, 16S rRNA high-throughput sequencing was performed on 20 root-associated soil samples. A total of 1,738,740 high-quality sequences were obtained. After clustering at a 97% sequence similarity threshold, 13,442 operational taxonomic units (OTUs) were identified, encompassing 45 phyla, 622 families, and 1224 genera. At the phylum level, 16 bacterial phyla exhibited relative abundances exceeding 1%. The dominant phyla included *Actinobacteriota* (26.1%), *Proteobacteria* (20.3%), *Acidobacteriota* (19.8%), and *Chloroflexi* (12.8%) (Figure 2A). At the generic level, 59 bacterial genera had relative abundances greater than 1%. The most prevalent genera included *RB41*, *Gaiella*, *Bradyrhizobium*, *Rhodanobacter*, *Pseudolabrys*, *Mycobacterium*, and *Arthrobacter*. Notably, samples Sc4, Sc5, and Sc14 exhibited similar microbial profiles, with *Rhodanobacter* showing a relative abundance ranging from 6.0% to 12.2% (Figure 2B), indicating potential regional or environmental consistency in microbial community structure.

**Figure 3:**
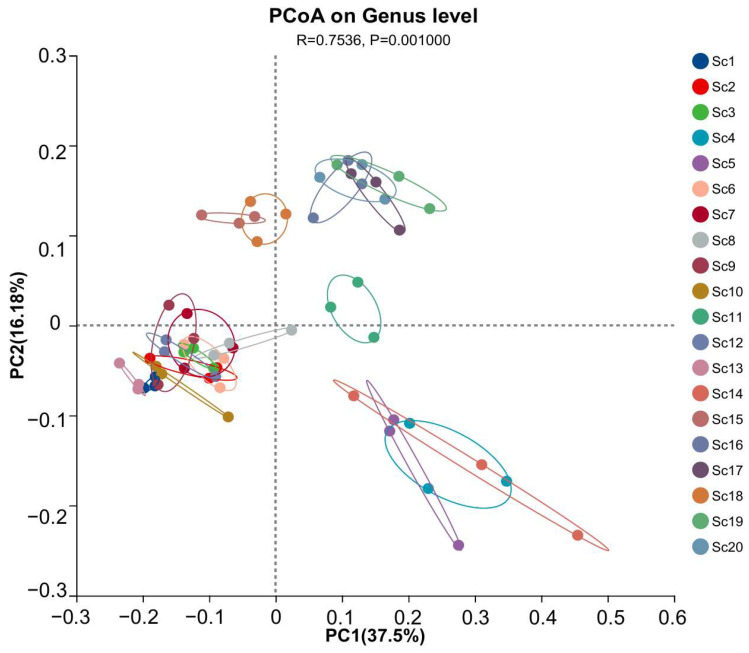
Principal coordinates analysis (PCoA) of rhizosphere microbial communities associated with different *S. chinensis* resources. Sc1–Sc20: *S. chinensis* resources. Clustering patterns reflect differences in microbial composition. The analysis was performed at the genus level. PC1 and PC2 explain 37.5% and 16.18% of the total variation, respectively. Statistical significance was assessed using PERMANOVA (R = 0.7536, *p* = 0.001).

To further assess compositional differences among samples, principal coordinates analysis (PCoA) was conducted. The results, as shown in Figure 3, indicate that samples collected from the same geographic region clustered closely, suggesting spatial homogeneity in rhizosphere microbial communities. Specifically, samples Sc4, Sc5, and Sc14 displayed high similarity in microbial composition, whereas sample Sc19 exhibited a distinct microbial profile, diverging significantly from the other samples. This observation aligns with the taxonomic composition analysis, reinforcing the influence of local environmental factors on rhizosphere microbial diversity.

### 3.5. Correlation Analysis Between Dominant Bacterial Genera, Soil Properties, and Lignan Components

**Figure 4:**
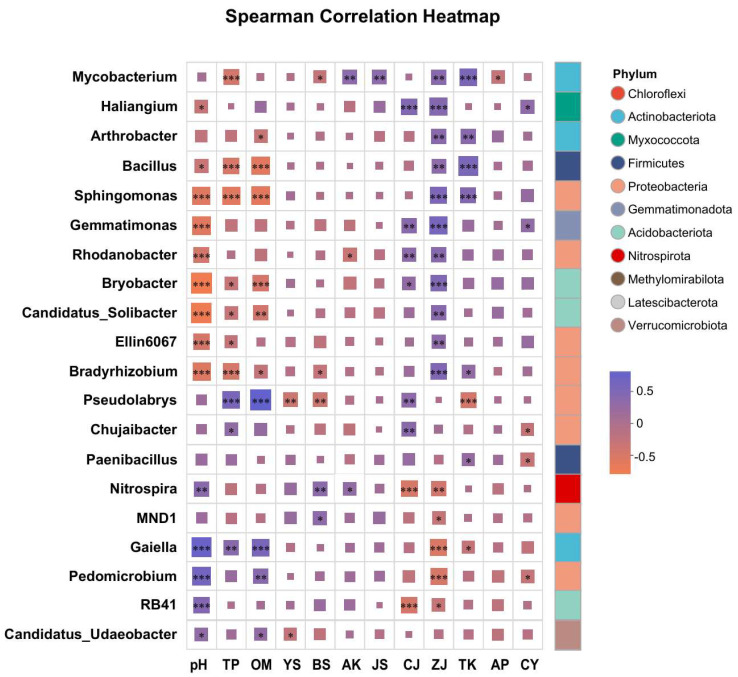
Spearman correlation analysis of lignan-associated rhizosphere microorganisms with soil physicochemical properties and lignan components in *S. chinensis*. TP: total phosphorus; TK: total potassium; TN: total nitrogen; AN: available nitrogen; AP: available phosphorus; AK: available potassium; OM: soil organic matter; pH: soil pH; CJ: schisandrol A; CY: schisandrol B; ZJ: schisantherin A; JS: schisandrin A; YS: schisandrin B; BS: schisandrin C. TL: total lignans. Significant correlations are indicated by asterisks: * *p* < 0.05, ** *p* ≤ 0.01, *** *p* ≤ 0.001.

At the genus level, taxonomic identification was performed on the top 50 most abundant operational taxonomic units (OTUs), resulting in the clear classification of 20 dominant bacterial genera (Figure 4). These genera were affiliated with eight phyla: *Actinobacteriota*, *Myxococcota*, *Firmicutes*, *Proteobacteria*, *Gemmatimonadota*, *Acidobacteriota*, *Nitrospirota*, and *Verrucomicrobiota*. Correlation correlation analysis revealed that *Mycobacterium*, *Arthrobacter*, *Haliangium*, *Bacillus*, *Sphingomonas*, *Rhodanobacter*, *Ellin6067*, *Bradyrhizobium*, *Pseudolabrys*, *Chujaibacter*, *Gemmatimonas*, *Bryobacter*, *MND1*, *Candidatus Sollbacter*, and *Nitrospira* were significantly or highly significantly positively correlated with lignan components. In contrast, *Gaiella*, *Paenibacillus*, *RB41*, and *Candidatus_Udaeobacter* showed significant or highly significant negative correlations with lignan accumulation.

Moreover, the relative abundance of these genera was significantly influenced by soil pH, total phosphorus (TP), and organic matter (OM), suggesting that soil nutrient conditions play a key role in shaping microbial community structure and may indirectly affect lignan biosynthesis in *S*. *chinensis*.

## 4. Discussion

This study provides a comprehensive analysis of rhizosphere soil physicochemical properties, fruit lignan content, and microbial community structure of *S*. *chinensis* across multiple cultivation sites. The findings reveal intricate and dynamic relationships among soil nutrient status, microbial diversity, and the biosynthesis of medicinal compounds, particularly lignans. Key soil physicochemical parameters, including pH, OM, TP, and TK, exert significant influence on lignan accumulation. Variations in these soil factors were closely associated with differences in the concentration of major lignan compounds such as schisandrol A, schisantherin A, and schisandrin B. These observations are consistent with previous studies indicating that soil fertility and nutrient availability play critical roles in secondary metabolite production in medicinal plants [1,26,27].

Importantly, this study advances current understanding by integrating soil nutrient profiles with rhizosphere microbial community data to explore their combined influence on lignan biosynthesis. While earlier research has separately examined soil conditions or microbial effects, the integrated soil–microbe–metabolite framework presented here offers a novel perspective [36,37]. As core indicators of soil fertility, physicochemical properties not only support plant growth but also shape microbial communities and regulate metabolic pathways [38,39,40]. Numerous studies have demonstrated strong correlations between soil nutrients and secondary metabolites in medicinal plants, such as anthraquinones in *Rheum officinale* [41], polysaccharides and saponins in *Polygonatum kingianum* [42], and ginsenosides in *Panax ginseng* [43]. In alignment with these findings, our results show that lignan content in *S. chinensis* is significantly correlated with OM, pH, available phosphorus (AP), and total nitrogen (TN), reinforcing the hypothesis that soil conditions indirectly regulate metabolite accumulation via microbial mediation [44].

The microbial analysis identified 45 phyla and 1224 genera, with dominant taxa including Proteobacteria, Actinobacteriota, Acidobacteriota, and Chloroflexi—consistent with rhizosphere profiles of other economically important crops [45,46]. Among the 59 genera with relative abundance exceeding 1%, Bacillus and Stenotrophomonas were notably enriched in samples from the Zhashui region, echoing previous reports [36]. These genera, along with others such as Arthrobacter and Paenibacillus, have been shown to enhance biomass and active compound accumulation in medicinal plants [47,48,49,50,51,52]. Their presence and abundance patterns suggest potential functional roles in regulating lignan biosynthesis, aligning with our hypothesis that site-specific microbial communities contribute to metabolite variation.

## 5. Conclusions

This study systematically investigated the rhizosphere soil physicochemical properties, fruit lignan content, and microbial community structure of *Schisandra chinensis* across different cultivation sites. The results demonstrated that potassium fertilization significantly promoted lignan accumulation, whereas nitrogen fertilization had a pronounced inhibitory effect. Soil pH, organic matter (OM), total phosphorus (TP), and total potassium (TK) were found to strongly influence the distribution of rhizosphere microbial communities. Moreover, dominant bacterial genera—including *Mycobacterium*, *Arthrobacter*, *Bradyrhizobium*, and *Rhodanobacter*—exhibited strong correlations with lignan content and were notably affected by soil nutrient conditions.

These findings underscore the indispensable roles of both soil fertility and rhizosphere microbial composition in shaping the medicinal quality of *S. chinensis*. Future research should focus on functional validation of key microbial taxa and elucidation of their mechanistic contributions to lignan biosynthesis. Such insights will provide a scientific foundation for targeted soil management and microbial interventions aimed at enhancing fruit quality and supporting sustainable cultivation practices.

## Figures and Tables

**Table 1:** Origin of the samples.

Sample ID	Region	Coordinates	Sample ID	Region	Coordinates
Sc1	Jingyu, Jilin	42.22° N, 126.85° E	Sc11	Jingyu, Jilin	42.25° N, 126.95° E
Sc2	Jingyu, Jilin	42.39° N, 126.92° E	Sc12	Jingyu, Jilin	42.36° N, 126.92° E
Sc3	Jingyu, Jilin	42.32° N, 126.99° E	Sc13	Jingyu, Jilin	42.40° N, 126.91° E
Sc4	Jingyu, Jilin	42.19° N, 126.99° E	Sc14	Jingyu, Jilin	42.28° N, 127.01° E
Sc5	Jingyu, Jilin	42.31° N, 126.95° E	Sc15	Zhashui, Shaanxi	33.52° N, 109.15° E
Sc6	Jingyu, Jilin	42.30° N, 126.95° E	Sc16	Zhashui, Shaanxi	33.49° N, 109.15° E
Sc7	Jingyu, Jilin	42.25° N, 126.96° E	Sc17	Zhashui, Shaanxi	33.54° N, 109.12° E
Sc8	Jingyu, Jilin	42.28° N, 127.01° E	Sc18	Zhashui, Shaanxi	33.69° N, 109.20° E
Sc9	Jingyu, Jilin	42.20° N, 126.97° E	Sc19	Zhashui, Shaanxi	33.51° N, 109.12° E
Sc10	Jingyu, Jilin	42.29° N, 126.95° E	Sc20	Zhashui, Shaanxi	33.53° N, 109.12° E

**Table 2:** High-performance liquid gradient elution condition.

Time (t/s)	Velocity of Flow (mL/min)	A (%)	B (%)
0	1.0	45	55
20	1.0	25	75
40	1.0	22	78
45	1.0	22	78
47	1.0	5	95
52	1.0	5	95
55	1.0	45	55
60	1.0	45	55

**Table 3:** Analysis of physicochemical properties of rhizosphere soil in *S*. *chinensis*.

Cultivation Sites (n = 3)	pH	OM (%)	TP (mg/Kg)	AP (mg/Kg)	TK (mg/Kg)	AK (mg/Kg)	TN (mg/Kg)	AN (mg/Kg)
Sc1	6.46 ± 0.11 a	15.6 ± 0.36 b	2209.27 ± 24.25 bcd	54.31 ± 0.99 f	12,856.6 ± 353.67 def	89.23 ± 1.43 de	6989.75 ± 470.1 b	514.64 ± 13.58 bc
Sc2	6.05 ± 0.1 cde	11.73 ± 0.13 de	2357.41 ± 45.31 ab	133.18 ± 3.2 c	12,965.66 ± 400.4 cdef	43.43 ± 0.61 ghi	5542.25 ± 167.12 cd	436.8 ± 16.8 de
Sc3	5.8 ± 0.08 ef	7.57 ± 0.15 h	1976.85 ± 29.49 bcdef	157.01 ± 2.73 b	13,069.11 ± 438.22 cdef	32.28 ± 1.03 i	3466.88 ± 179.25 g	266.56 ± 17.24 i
Sc4	6.37 ± 0.02 ab	11.52 ± 0.25 de	1689 ± 15.44 cdefg	25.78 ± 3.01 jk	13,944.34 ± 440.03 bcdef	36.37 ± 1.09 hi	5216.79 ± 153.26 de	388.64 ± 42.55 efg
Sc5	5.86 ± 0.3 def	11.44 ± 0.63 de	2263.85 ± 895.6 bc	70.93 ± 3.9 e	14,505.34 ± 71.65 bcde	84.08 ± 1.27 de	5144.39 ± 578.1 def	446.88 ± 34.47 d
Sc6	5.85 ± 0.01 def	12.14 ± 0.43 d	1353.73 ± 33.88 fg	22.33 ± 0.55 k	14,702.99 ± 602.7 bcd	42.46 ± 0.97 ghi	5687.56 ± 114.14 cd	463.68 ± 7.7 cd
Sc7	6.13 ± 0.04 bcd	9.03 ± 0.58 g	1299.27 ± 52.61 g	10.03 ± 1.31 l	14,323.76 ± 315.24 bcde	244.3 ± 17.51 a	4561 ± 114.74 f	347.20 ± 18.51 fgh
Sc8	6.02 ± 0.01 de	24.55 ± 0.86 a	1828.82 ± 30.21 bcdefg	46.83 ± 5.14 fg	12,995.07 ± 306.12 cdef	161.54 ± 1.9 b	11,371.56 ± 165.04 a	688.24 ± 13.05 a
Sc9	6.38 ± 0.02 ab	10.74 ± 0.22 ef	1457.45 ± 17.9 efg	23.15 ± 1.2 k	14,160.45 ± 449.49 bcdef	122.06 ± 1.76 c	5046.32 ± 138.57 def	336.56 ± 3.5 gh
Sc10	6.34 ± 0.02 abc	9.77 ± 0.12 fg	1592.32 ± 35.89 defg	46.19 ± 6.15 fg	13,436.85 ± 346.94 cdef	113.78 ± 2.44 c	4735.23 ± 93.8 ef	333.76 ± 11.19 h
Sc11	5.27 ± 0.05 i	23.69 ± 0.68 a	1976.76 ± 67.06 bcdef	69.03 ± 1.45 e	10,435.99 ± 505.67 g	48.25 ± 1.37 gh	10,906.8 ± 156.98 a	533.68 ± 15.24 b
Sc12	5.86 ± 0.05 def	13.37 ± 0.2 c	1996.15 ± 37.58 bcde	69.64 ± 2.47 e	12,734.5 ± 930.17 def	52.83 ± 3.83 g	5954.34 ± 332.6 c	393.12 ± 8.89 ef
Sc13	6.14 ± 0.02 bcd	14.72 ± 0.17 b	1992.82 ± 35.65 bcde	26.23 ± 1.56 jk	12,237.94 ± 308.34 fg	95.00 ± 3.18 d	7422.4 ± 140.85 b	533.12 ± 13.99 b
Sc14	5.39 ± 0.08 hi	9.6 ± 0.11 fg	2973.87 ± 32.68 a	299.16 ± 3.27 a	12,552.12 ± 308.08 ef	67.58 ± 3.1 f	4560.08 ± 119.66 f	359.8 ± 8.74 fgh
Sc15	5.86 ± 0.19 def	3.3 ± 0.28 ij	1411.22 ± 65.82 efg	89.23 ± 1.43 d	13,482.83 ± 1367.12 cdef	89.72 ± 1.89 de	1476.3 ± 10.07 h	120.68 ± 19.34 j
Sc16	5.79 ± 0.07 ef	2.98 ± 0.09 j	1247.62 ± 45.81 g	43.43 ± 0.61 gh	13,102.11 ± 221.67 cdef	77.64 ± 6.01 ef	1450.93 ± 10.41 h	119.00 ± 6.42 j
Sc17	5.57 ± 0.04 fgh	2.84 ± 0.19 j	1237.15 ± 54.88 g	32.28 ± 1.03 ij	14,898.4 ± 1621.75 bc	68.83 ± 2.18 f	1395.72 ± 33.9 h	126.00 ± 19.81 j
Sc18	5.77 ± 0.05 ef	2.98 ± 0.33 j	1263.7 ± 32.01 g	36.37 ± 1.09 hi	15,791.65 ± 647.39 ab	69.48 ± 0.72 f	1415.19 ± 38.98 h	114.24 ± 4.44 j
Sc19	5.76 ± 0.04 efg	4.29 ± 0.3 i	1379.72 ± 41.36 efg	84.08 ± 1.27 d	17,536.44 ± 247.9 a	116.14 ± 5.28 c	1981.05 ± 34.07 h	146.72 ± 5.4 j
Sc20	5.47 ± 0.07 ghi	4.44 ± 0.12 i	1325.6 ± 47.91 g	42.46 ± 0.97 gh	17,646.84 ± 513.83 a	76.23 ± 1.94 ef	1619.38 ± 9.25 h	126.00 ± 4.2 j
CV(%)	5.91	59.54	28.32	93.48	12.69	56.35	59.65	49.42

Note: Values are presented as mean ± standard deviation (SD), based on three replicates (n = 3). OM: Organic Matter; TP: Total Phosphorus; AP: Available Phosphorus; TK: Total Potassium; AK: Available Potassium; TN: Total Nitrogen; AN: Alkali-hydrolyzable Nitrogen. Statistical differences among sites were assessed using one-way ANOVA followed by Tukey’s post hoc test. Different letters in this table indicate significant differences among samples (*p* < 0.05).

**Table 4:** Analysis of lignan content in *S. chinensis* fruits.

Cultivation Sites (n = 3)	Total Lignans (mg/g)	Schisandrol A (mg/g)	Schisandrol B (mg/g)	Schisantherin A (mg/g)	Schisandrin A (mg/g)	Schisandrin B (mg/g)	Schisandrin C (mg/g)
Sc1	9.05 ± 0.35 i	4.85 ± 0.05 klm	1.45 ± 0.12 gh	0.43 ± 0.04 e	0.73 ± 0.10 g	1.29 ± 0.09 ij	0.29 ± 0.01 ij
Sc2	8.99 ± 0.351 i	4.61 ± 0.05 lm	1.26 ± 0.09 gh	0.40 ± 0.03 e	0.29 ± 0.03 h	2.04 ± 0.15 g	0.39 ± 0.02 gh
Sc3	12.96 ± 0.21 fg	6.09 ± 0.11 ghi	1.05 ± 0.02 hi	0.48 ± 0.05 de	0.77 ± 0.02 fg	4.07 ± 0.04 d	0.50 ± 0.01 f
Sc4	17.85 ± 0.41 bc	7.82 ± 0.12 bcd	3.90 ± 0.04 b	0.67 ± 0.30 cde	1.39 ± 0.04 cd	3.40 ± 23.74 e	0.67 ± 0.00 e
Sc5	14.35 ± 1.18 ef	8.58 ± 1.07 ab	1.09 ± 0.05 hi	1.37 ± 0.06 b	1.42 ± 0.03 cd	1.73 ± 0.02 ghi	0.16 ± 0.01 mn
Sc6	11.17 ± 0.481 h	5.69 ± 0.08 hij	2.30 ± 0.19 f	0.74 ± 0.07 cde	0.71 ± 0.09 g	1.47 ± 0.10 hij	0.25 ± 0.02 jk
Sc7	16.15 ± 0.24 cd	6.78 ± 0.09 efg	1.63 ± 0.03 g	0.58 ± 0.06 cde	1.25 ± 0.09 cde	5.17 ± 0.04 c	0.74 ± 0.01 d
Sc8	17.12 ± 0.37 c	8.03 ± 0.18 bc	2.81 ± 0.03 de	1.38 ± 0.11 b	1.78 ± 0.06 b	2.91 ± 0.08 ef	0.22 ± 0.00 klm
Sc9	11.34 ± 0.15 gh	5.77 ± 0.10 hij	0.73 ± 0.04 i	0.50 ± 0.04 de	2.45 ± 0.05 a	1.60 ± 0.03 ghi	0.29 ± 0.00 ij
Sc10	13.32 ± 0.13 ef	6.27 ± 0.02 fgh	1.27 ± 0.05 gh	0.48 ± 0.26 de	1.82 ± 0.01 b	3.14 ± 60.02 ef	0.34 ± 0.01 hi
Sc11	12.97 ± 0.10 fg	6.93 ± 0.03 ef	3.40 ± 0.05 c	0.80 ± 0.03 cd	0.99 ± 0.05 efg	0.67 ± 0.06 k	0.17 ± 0.01 lmn
Sc12	22.70 ± 0.28 a	9.11 ± 0.08 a	3.24 ± 0.11 cd	0.68 ± 0.02 cde	1.43 ± 0.01 c	6.96 ± 0.11 a	1.27 ± 0.02 b
Sc13	14.17 ± 0.24 ef	6.27 ± 0.10 fgh	3.19 ± 0.00 cd	0.44 ± 0.02 e	1.08 ± 0.06 def	2.68 ± 0.06 f	0.50 ± 0.00 f
Sc14	16.48 ± 0.46 cd	8.29 ± 0.29 ab	1.22 ± 0.01 gh	0.58 ± 0.07 cde	0.77 ± 0.10 fg	4.95 ± 0.14 c	0.67 ± 0.02 e
Sc15	14.76 ± 0.91 de	4.48 ± 0.12 lm	1.25 ± 0.09 gh	0.62 ± 0.06 cde	1.33 ± 0.29 cd	6.21 ± 0.42 b	0.86 ± 0.05 c
Sc16	11.32 ± 052 gh	5.44 ± 0.06 ijk	2.21 ± 0.17 f	0.72 ± 0.07 cde	0.85 ± 0.12 fg	1.87 ± 0.13 gh	0.23 ± 0.01 jkl
Sc17	18.89 ± 0.95 b	7.39 ± 0.12 cde	3.03 ± 0.25 cd	0.56 ± 0.05 cde	1.37 ± 0.19 cd	5.17 ± 0.37 c	1.37 ± 0.06 a
Sc18	14.21 ± 0.84 ef	4.34 ± 0.14 m	4.81 ± 0.38 a	0.88 ± 0.08 c	0.72 ± 0.14 g	2.96 ± 0.21 ef	0.50 ± 0.00 f
Sc19	14.18 ± 0.78 ef	5.25 ± 0.05 jkl	2.82 ± 0.22 de	0.73 ± 0.07 cde	0.99 ± 0.14 efg	3.94 ± 0.29 d	0.46 ± 0.02 fg
Sc20	13.91 ± 0.64 ef	7.18 ± 0.18 de	2.54 ± 0.20 ef	2.24 ± 0.22 a	0.78 ± 0.10 fg	1.03 ± 0.07 jk	0.13 ± 0.01 n
CV(%)	23.00	22.11	49.07	57.84	43.32	56.01	68.32

Note: Values are expressed as mean ± standard deviation (SD), based on three replicates (n = 3). The lignans measured include Schisandrol A, Schisandrol B, Schisantherin A, Schisantherin B, Schisandrin A, Schisandrin B, and Schisandrin C. Statistical differences among sites were assessed using one-way ANOVA followed by Tukey’s post hoc test. Different letters in this table indicate significant differences among samples (*p* < 0.05).

## Data Availability

16S rRNA sequencing raw data can be downloaded at the National Genomics Data Center (NGDC), (https://ngdc.cncb.ac.cn/gsub/submit/bioproject/PRJCA045516) (accessed on 1 September 2025) when the article is published. The data presented in this study are available on request from the corresponding author.
